# Unilateral Cervical Vagotomy Modulates Immune Cell Profiles and the Response to a Traumatic Brain Injury

**DOI:** 10.3390/ijms23179851

**Published:** 2022-08-30

**Authors:** M. Karen Newell-Rogers, Amanda Duong, Rizwan Nazarali, Richard P. Tobin, Susannah K. Rogers, Lee A. Shapiro

**Affiliations:** 1 School of Medicine, Texas A&M University, 8447 Riverside Parkway, Bryan, TX 77807, USA; 2 BCell Solutions, Inc., Colorado Springs, CO 80907, USA; 3 Department of Anesthesiology, School of Medicine, University of Colorado, Denver, CO 80309, USA; 4 Department of Surgery-Surgical Oncology, School of Medicine, University of Colorado Anschutz Medical Campus, Aurora, CO 80045, USA

**Keywords:** immune system, cholinergic anti-inflammatory pathway, spleen, splenocytes, B cells, T cells, vagus nerve

## Abstract

TBI induces splenic B and T cell expansion that contributes to neuroinflammation and neurodegeneration. The vagus nerve, the longest of the cranial nerves, is the predominant parasympathetic pathway allowing the central nervous system (CNS) control over peripheral organs, including regulation of inflammatory responses. One way this is accomplished is by vagus innervation of the celiac ganglion, from which the splenic nerve innervates the spleen. This splenic innervation enables modulation of the splenic immune response, including splenocyte selection, activation, and downstream signaling. Considering that the left and right vagus nerves have distinct courses, it is possible that they differentially influence the splenic immune response following a CNS injury. To test this possibility, immune cell subsets were profiled and quantified following either a left or a right unilateral vagotomy. Both unilateral vagotomies caused similar effects with respect to the percentage of B cells and in the decreased percentage of macrophages and T cells following vagotomy. We next tested the hypothesis that a left unilateral vagotomy would modulate the splenic immune response to a traumatic brain injury (TBI). Mice received a left cervical vagotomy or a sham vagotomy 3 days prior to a fluid percussion injury (FPI), a well-characterized mouse model of TBI that consistently elicits an immune and neuroimmune response. Flow cytometric analysis showed that vagotomy prior to FPI resulted in fewer CLIP+ B cells, and CD4+, CD25+, and CD8+ T cells. Vagotomy followed by FPI also resulted in an altered distribution of CD11b^high^ and CD11b^low^ macrophages. Thus, transduction of immune signals from the CNS to the periphery via the vagus nerve can be targeted to modulate the immune response following TBI.

## 1. Introduction

Traumatic brain injury (TBI) occurs in millions of people in the United States each year [1]. The severity of TBIs can range from mild to severe. While more severe injuries often cause detrimental outcomes, even mild injuries have been linked to behavioral and neurological dysfunction, including cognitive decline, depression, addiction, anxiety, post-traumatic epilepsy, and an increased susceptibility to developing Alzheimer’s disease [2,3,4,5,6,7]. It has been estimated that the annual cost of TBI in the United States is as high as USD 20 billion [8]. This cost does not factor in the reduced quality of life often experienced by the patient, as well as the lives of friends, families, and loved ones. Despite this enormous toll, treatment strategies are lacking to treat post-TBI outcomes.

TBI often induces neuroinflammation and a peripheral immune response [9,10]. This includes expansion and activation of immune cell subsets, and extravasation of immune cells to and from the blood, spleen, liver, and gut [11,12,13]. Although the precise mechanisms by which a TBI in the central nervous system (CNS) induces a systemic immune response are not fully understood, several mechanisms have been identified. Inflammatory proteins such as cytokines and chemokines that are initially released in the CNS in response to the injury travel through the blood to signal peripheral immune responses [14,15]. TBI can also induce the release of acute phase response effector proteins and the subsequent activation of the hepatic acute phase response [16,17]. The acute phase response is an early component of the innate immune response that is involved in orchestrating immune response mechanisms [18]. In addition to paracrine signaling, studies also demonstrate that cranial nerve X, the vagus nerve, plays an important bidirectional role in the immune response by modulating the peripheral/neuroimmune axis [19,20,21,22].

There are several pathways that enable vagus nerve contributions to the immune response. Vagal innervation of the gut and liver allows modulation of the vast number of immune cells in these organs [23]. The abdomen, jejunum, ileum, and cecum are innervated by the right celiac branch of the vagus nerve and a vagotomy at this level abolishes intestinal inflammation. It was also shown that vagal afferents within the gut are important in the activation of the vagal efferent response to intestinal manipulation [24]. The vagus nerve, along with the spleen, is also prominently involved in the cholinergic anti-inflammatory pathway (CAP) [25,26]. The CAP is an autonomic pathway that provides a mechanism whereby the CNS can receive afferent information about inflammatory stimuli and regulate the immune response via efferent projections [20,21]. Thus, the vagus nerve provides an anatomical link between the periphery and the CNS that enables the CNS to receive information about immune status and inflammation and, in turn, modulate ongoing immune responses.

In order to modulate splenocyte selection, activation, and downstream signaling, the axon terminals of vagal efferents release acetylcholine (ACh) within the celiac ganglion [27,28]. The splenic nerve, originating from the celiac ganglion, innervates the spleen, releasing norepinephrine (NE) that binds to beta-adrenergic receptors on a special type of choline acetyltransferase-expressing T cells (T_ChAT_). These T_ChAT_ cells in the spleen use choline acetyltransferase to synthesize and release ACh [29], activating the α-7 nicotinic acetylcholine receptor (α7 nAChR) on macrophages [30]. This attenuates macrophage activation and subsequently inhibits the release of TNFα [29,31], a major pro-inflammatory cytokine, along with other pro-inflammatory cytokines including IL-1β and IL-6 [25,26,27]. There are also a small number of sensory afferents that innervate the spleen, providing the CNS with vital information on the inflammatory state [32]. Studies have shown that antibody production and peripheral TNFα levels are impaired if the ascending or descending vagus nerve pathways are blocked [33,34]. Therefore, the vagus nerve uses afferent and efferent input to modulate the immune response, which includes influencing cell selection and activation in the spleen via the splenic nerve.

The extent to which the vagus nerve influences the splenic immune response to a CNS injury is less understood. Splenectomy in stroke and TBI models has been shown to be advantageous to lesion size and functional outcomes [35,36], and inhibiting peripheral lymphocyte activation after a TBI has been shown to improve functional and neuroanatomical parameters [37]. Whereas the effect of splenectomy has been tested after TBI, the cellular immune response to vagotomy has not been examined in this context. Importantly, vagus nerve stimulation is known to be anti-inflammatory and anti-neuroinflammatory, demonstrating a clear immunomodulatory effect of manipulating this pathway. It is thought that the anti-inflammatory action of vagus nerve stimulation may underlie the therapeutic efficacy of vagus nerve stimulation in treating epilepsy, depression, migraines, and other disorders [38,39]. Considering the importance of the vagus nerve at modulating inflammatory and neuroinflammatory responses, and its therapeutic uses, fully understanding the role of the vagus nerve in the immune response after a TBI could provide novel therapeutic avenues for eventual treatments. This is especially important because there are currently no treatments available that can reduce the incidence of post-TBI syndromes.

The current study assesses the effects of left versus right vagotomy on immune cell profiles and incorporates the lateral fluid percussion injury (FPI) model of TBI that consistently elicits an immune response [37,40,41,42,43] to test the hypothesis that a prior left unilateral vagotomy will alter FPI-induced immune cell selection and activation in the spleen.

## 2. Results

### 2.1. Left and Right Vagotomy Alters the Immune Profile of Macrophages, B Cells, and T Cells

**Figure 1:**
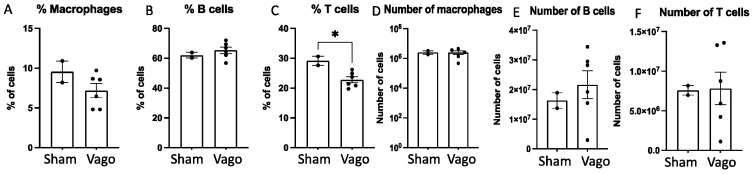
Vagotomy alters the splenic immune profile. Left and right unilateral vagotomy (Vago) groups were combined and their immune profiles were compared to sham vagotomy (Sham) mice. In (**A**), a modest decrease in percent macrophages was observed in the vagotomy group, but the overall number of macrophages (**D**) was unchanged. In (**B**,**E**), there were no significant changes to the percent or number of total B cells, respectively. In (**C**), the percent of T cells is significantly less in the vagotomy group, whereas in (**F**), the total number of T cells is not significantly different. Thus, there appears to be a shift in the distribution of splenic immune cells after vagotomy, with lower percentages of macrophages and T cells. * *p* < 0.03.

The left and right branches of the vagus nerve have different peripheral and immune organ innervation, and the vagus nerve can modulate the immune response by influencing splenocyte selection, activation, and downstream signaling [19,44,45]. However, the influence of the left and right vagus nerves on splenic immune cell selection has not been elucidated. Thus, we assessed the effects of left versus right vagotomy on the frequency of splenic macrophages, T cells, and B cells (Table 1). The results indicate that left or right vagotomy both similarly shift the immune profile. Since no significant differences between the left and right vagotomy were observed, we combined the data and compared them to sham vagotomy mice. The combined results demonstrate that left and right unilateral vagotomy causes a modest decrease in the percent of macrophages (Figure 1A), no change in the percent of total B cells (Figure 1B), and a significant decrease in the percent of T cells (Figure 1C; *p* < 0.03), relative to sham vagotomy. The overall number of splenic macrophages was unchanged (Figure 1D), whereas the B cells (Figure 1E) and T cells (Figure 1F) showed modest increases.

### 2.2. Left Vagotomy Prior to FPI Reduces Macrophages, B Cells, and CLIP^+^ B Cells

**Figure 2:**
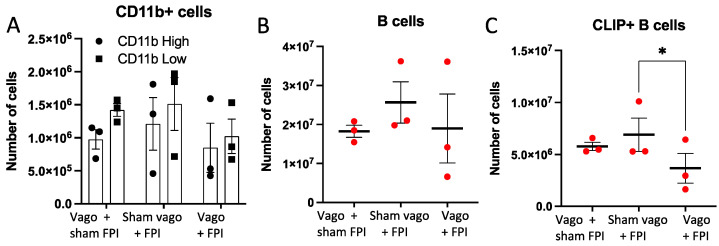
Influence of vagotomy on macrophages and B cells after FPI. In (**A**), comparison of the number of splenic macrophages stratified by high or low CD11b expression. No significant differences were observed between groups. In (**B**), quantification of the flow cytometric analysis of total splenic B cells also revealed no significant differences between groups. In (**C**), examination of CLIP+ B cells demonstrated that vagotomy prior to FPI (Vago + FPI) resulted in a significant decrease in CLIP+ B cells compared to sham vagotomy + FPI (Sham vago + FPI; * = *p* < 0.05).

Examination of the number of CD11b+ macrophages (Figure 2A) and CD19+ B cells (Figure 2B), did not reveal significant differences between groups. Chi Square analysis of CD11b^high^ and CD11b^low^ cells was performed and found significance (*p* < 0.0001, not shown), which suggests a change in the frequency of these two macrophage populations. Flow cytometric analysis of CLIP+ B cell subsets revealed a significant decrease in the number of these B cells in vagotomy + FPI mice compared to sham vagotomy + FPI mice (*p* = 0.0324, Figure 2C).

### 2.3. T Cell and T Cell Subsets Are Influenced by Vagotomy and FPI

**Figure 3:**
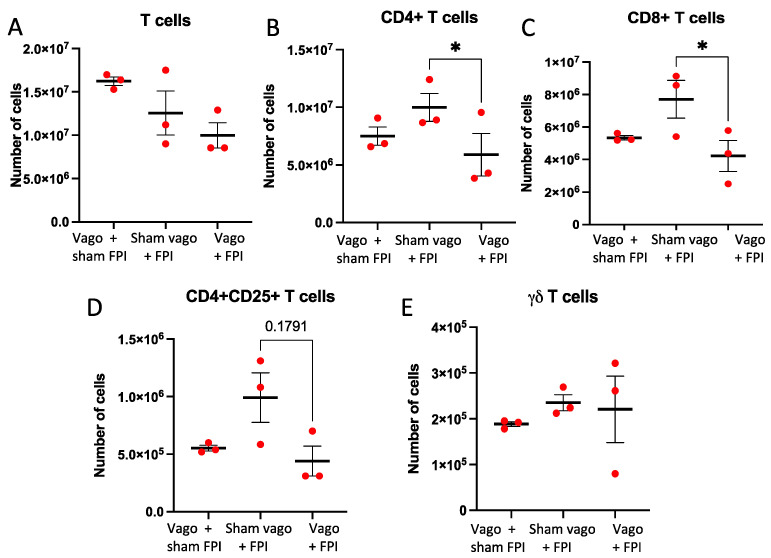
Vagotomy prevents FPI-induced expansion of specific T cell subsets. In (**A**), comparison of the number of CD3+ T cells following vagotomy + sham FPI (Vago + sham FPI), sham vagotomy + FPI (Sham vago + FPI), or vagotomy + FPI (Vago + FPI) showed no significant differences. In (**B**), examination of the number of CD4+ T cells (as stratified by expression of CD3+, CD19−, CD4+, CD8−) showed a significant decrease in CD4+ T cells in the Vago + FPI compared to the Sham vago + FPI group. In (**C**), a significant decrease in CD8+ T cells (as determined by cell surface expression of CD3+, CD19−, CD4−, CD8+) in Vago + FPI compared to Sham vago + FPI was observed. There was also a trend towards increased CD8+ T cells in Sham vago + TBI compared to Vago + sham FPI. In (**D**), examination of CD4+CD25+ T cells characterizing CD4+ T regulatory cells (Tregs), as determined by cell surface expression of CD3+, CD19−, CD4+, CD8−, indicates that Sham vago + FPI results in a trend towards increased anti-inflammatory Tregs relative to Vago + sham FPI and Vago + FPI. In (**E**), there were no significant differences observed for the number of CD3+γδ+ T cells (γδ T cells), as determined by cell surface expression of CD3+CD19-TCRγδ+; * = *p* < 0.05.

Examination of the overall number of CD3+ T cells demonstrated a trend towards a difference (*p* = 0.094), wherein vagotomy + FPI resulted in less T cells compared to vagotomy + sham FPI (Figure 3A). Significantly less CD4+ T cells were observed in the vagotomy + FPI group, compared to sham vagotomy + FPI (*p* < 0.05; Figure 3B). A trend approaching significance (*p* = 0.055) was also identified, showing increased T cells in the sham vagotomy + FPI compared to the vagotomy + sham FPI (Figure 3B). Analysis of CD8+ T cells showed that FPI + vagotomy caused a significant decrease in CD8 + T cells (*p* < 0.03) compared to sham vagotomy + FPI (Figure 3C). Thus, it is possible that FPI induces an increase in CD4+ and CD8+ T cells, and the vagus nerve is involved in either the expansion and/or the extravasation of these T cells following TBI. Quantification of the number of CD4+CD25+ T cells revealed a trend (*p* = 0.179) towards an increased number of cells in sham vagotomy + FPI compared to vagotomy + FPI (Figure 3D). No significant changes were observed for γδ T cells (Figure 3E).

## 3. Discussion

In the current study, the impact of left versus right vagotomy on the splenic immune profile three days after vagotomy was assessed, as was the splenic immune response to a left unilateral vagotomy 3 days prior to a TBI. The results showed that both a left and a right unilateral vagotomy had similar effects on splenic lymphocytes. A left vagotomy was chosen for examination prior to FPI because the right branch has greater cardiac innervation, and the left vagus nerve is typically used in therapeutic applications [46,47,48,49]. The results showed that a vagotomy prior to a TBI prevented the TBI-induced expansion of CLIP+ B cells and several T cell subsets.

While the spleen has been shown to play a major role in the peripheral immune response to a TBI, the contributions of the spleen and other peripheral immune components to a CNS injury continue to be elucidated [50,51,52]. Splenectomy immediately following TBI reduced pro-inflammatory signals via NF-kB, improved cognitive function, and decreased mortality after severe TBI in rats [35,36]. Conversely, in humans, a splenectomy prior to a TBI resulted in increased mortality [53]. Studies in other CNS injury models, including stroke, have also demonstrated the ability of the spleen to modulate functional and physiological outcomes [51,52,54,55,56]. Thus, the splenic immune response is capable of both improving or exacerbating outcomes to brain injuries, and a splenectomy does not appear to be a desirable clinical therapy [57]. Alternatively, vagus nerve manipulations have the potential to selectively target immune outcomes in a number of neurological and neurodegenerative disorders and have been demonstrated to be highly tolerable [47,48,49,58,59].

The observation that a vagotomy prior to the TBI significantly reduced splenic CD8+ T cells and showed a trend towards reducing regulatory T cells (CD4+/CD25+) is consistent with a dual role for the splenic immune response after injury. It was noted that a protracted increase in effector/memory CD8+ T cells (expressing granzyme B) in the injured brain was associated with progressive neurological/motor impairment, increased circulating brain-specific autoantibodies, and myelin-related pathology. Genetic deficiency or antibody depletion of CD8+ T cells was neuroprotective and improved neurological outcomes [60]. Other studies demonstrated that TBI caused astrocyte activation resulting in production of IL15 that led to CD8+ T cell activation and neuronal apoptosis [61]. Conversely, the frequency of circulating Treg cells has been shown to positively correlate with better clinical outcome following TBI [62]. Our data demonstrate that when vagotomy precedes FPI, the expansion of CD8+ T cells and Treg cells is reduced. Thus, vagotomy prior to FPI results in a reduction in both pro- and anti-inflammatory cells, possibly underscoring mixed results in preclinical and clinical settings.

One of the major pathways in splenic immune regulation is the CAP. This reflex immune pathway provides an anatomical substrate for the CNS to regulate peripheral inflammation [26,28,32]. A previous study found B and T cell expansion at 24 h after FPI. This expansion included CD11b+ macrophages and B and T cell subsets, including CD3+, CD4+, and CD8+ T cells, Treg cells, and CLIP+ B cells [37]. It was also observed that an antibody to CLIP was neuroprotective and reversed the FPI-induced expansion of T and CLIP+ B cells [37]. That vagotomy prior to the FPI also appears to have prevented the FPI-induced increase in T and CLIP+ B cells suggests that an intact vagus nerve is needed to transduce the brain-injury-induced immune signal to the spleen. There are several other possible interpretations of this data. One seemingly counterintuitive conclusion is that FPI is anti-inflammatory after vagotomy. A second possible conclusion is that the vagotomy 3 days prior to FPI causes T and CLIP+ B cell extravasation, resulting in a depleted immune response to the subsequent FPI. It would be interesting to examine the splenic immune response in mice given FPI more than 3 days after the vagotomy to see how the response differs from the current study. A third possible interpretation is that vagotomy has a priming or compensatory effect on the contralateral vagus nerve, the net contralateral effect being hyper-activation of the CAP in response to inflammatory stimuli such as FPI. A fourth possible interpretation is that the left and right vagus nerve counteract each other’s effects on the spleen in a “push/pull” manor. There is a precedent for this possibility in other cranial nerves. For example, a unilateral lesion of cranial nerve XII results in a contralateral hyper-excitatory effect on the genioglossus tongue muscles, because the lesion removed the inhibition by the contralateral nerve [63]. Although our comparison of left and right vagotomy do not support this latter conclusion, it is possible that an immune stimulus is needed to fully assess the independent influence of the left and right vagus nerves on the splenic immune response. Follow-up studies are needed to address these very intriguing possibilities.

## 4. Materials and Methods

### 4.1. Cervical Vagotomy

For these experiments, 8-week-old, male, C57BL/6 (*n* = 6) mice were used. The mice were randomly assigned to either the left or right vagotomy or left or right sham vagotomy group. On the day of the procedures, all mice were kept in a separate room unless they were undergoing the procedure to decrease the impact of a potential sympathetic fight or flight response. Initially, mice were put in a holding tank where isoflurane was infused to achieve sedation and were then shaved to prepare the area for incision. Thereafter, mice were given an intraperitoneal (i.p.) injection of a partial mu-opioid agonist, buprenorphine, as an analgesic. Finally, they were placed on continuous isoflurane sedation and immobilized under a microscope. The initial incision was made just left of the midline. The fascia was separated and dissected using scissors. The bi-lobed submandibular salivary glands were identified and were bisected to visualize deeper structures. The left sternocleidomastoid was identified and securely pulled laterally. This revealed the carotid sheath which was subsequently pierced. The left vagus nerve was isolated from the left carotid artery and was transected. Sham animals underwent the identical procedure except the vagus nerve was not severed. Closure was performed with simple 2-0 Prolene sutures with application of antibiotic ointment. Mice were placed in isolated cages, with a heating pad underneath, to recover from anesthesia before being placed back with cage mates. Mice were routinely checked twice daily post-operatively, A.M. and P.M., to assess for signs of stress, wound infection, and weight.

### 4.2. Fluid Percussion Injury

Mice were assigned to fluid percussion injury (FPI) or sham at 72 h after the vagotomy or sham vagotomy. FPI was performed as previously described [37,40,64]. Briefly, prior to being placed in the stereotactic instrument, mice were anesthetized with isoflurane and shaven [37]. A 2 mm craniotomy over the left parietal cortex (antero-posterior: +1.5 mm; medio-lateral: −1.2 mm) was performed, after which the female end of a Luer-lock syringe was cemented over the craniotomy and attached to the fluid percussion apparatus (Custom Design & Fabrication, Inc., Sandston, VA, USA). FPI mice received a 12–16 ms FPI at a pressure of 1.4–1.6 atm; sham mice were connected to the FPI apparatus but no pressure pulse was delivered. The wound was closed using suture and the mice were returned to a heating pad in their cage. Food and water were available ad libitum and food mash was also provided after surgery. Mice were euthanized 24 h after the FPI, and the spleen was rapidly dissected.

### 4.3. Flow Cytometry

To characterize subsets of immune cells, the spleens were removed and single cell suspensions were prepared for flow cytometry, as previously described [37]. Once the tissues were dissociated into single cell suspensions, red blood cells were depleted. The remaining white cells were incubated with FC Block (BD Bioscience) to prevent non-specific binding of the staining antibodies via Fc receptors. The splenocytes were then stained with the following antibodies: CD3, CD4, CD8, CD25, CLIP, CD19, and CD11b. The Life Technologies LIVE/DEAD* Fixable Aqua Dead Cell Stain Kit was used to assess live cells according to the manufacturer’s directions. BD FACS Canto II flow cytometer was used to analyze the splenocytes and FlowJo software was used to analyze the data collected.

### 4.4. Statistical Analysis

Data were analyzed using GraphPad Prism Software version 9.4.0. For comparisons of two groups, unpaired Student’s T Tests were used to determine statistical significance with a significance cut-off of *p* < 0.05. A Chi-square test was also performed with a significance cut-off of *p* < 0.05. For comparisons between three or more groups, a repeated measures one-way ANOVA was used with Tukey least significant differences post hoc testing and with a significance cut-off of *p* < 0.05.

## 5. Conclusions

The results from this study indicate that the vagus nerve is involved in transducing FPI-induced inflammatory signals from the CNS to the spleen. Thus, manipulating the vagus nerve may provide an opportunity to modulate the splenic immune response to CNS injury. While vagotomy 3 days prior to FPI clearly prevented the FPI-induced expansion of T cell subsets and CLIP+ B cells, additional studies are needed to fully understand the causes and implications of this finding. It would be interesting to determine if vagotomy prior to FPI was neuroprotective or exacerbated neurodegeneration. Future studies are also needed to determine the potential antagonistic relationship between the left and right vagus nerve on the splenic immune response to CNS injury. These studies should further assess whether it is possible to functionally modulate the vagus nerve to control select immune cell expansion, activation, and extravasation as potential therapeutic targets following a CNS injury such as TBI.

## Figures and Tables

**Table 1:** Vagotomy alters the splenic immune profile following either right or left vagotomy. Quantification of flow cytometric analysis of the number of macrophages (CD11b+CD3−CD19−), B cells (CD19+CD3−), and T cells (CD3+CD19−), 24 h following vagotomy or sham vagotomy. No significant differences between left (L-Vagotomy) and right vagotomy (R-Vagotomy) were observed. Relative to sham vagotomy (Sham), both the left and right vagotomy resulted in similar B cell increases and macrophage and T cell decreases.

		Sham	L-Vagotomy	R-Vagotomy
**B Cells**	Mean	62.05	65.27	65.33
	SD	2.62	7.75	3.72
**Macrophages**	Mean	9.55	7.90	6.45
	SD	1.91	1.25	2.83
**T Cells**	Mean	29.10	22.23	23.33
	SD	2.12	3.48	1.78

## Data Availability

The data presented in this study are available in Unilateral cervical vagotomy modulates immune cell profiles and the response to a subsequent traumatic brain injury, and by request.
